# A Step Forward in Post-Mortem Interval Estimation: Multivariate Analysis of Ammonium, Albumin, and Potassium Levels in Vitreous Humor

**DOI:** 10.3390/diagnostics16131970

**Published:** 2026-06-24

**Authors:** Martina Focardi, Beatrice Defraia, Ilenia Bianchi, Barbara Gualco, Andrea Costantino, Rossella Grifoni, Alessandra Fanelli, Tiziana Biagioli, Costanza Bossi, Vilma Pinchi, Luisa Lanzilao

**Affiliations:** 1 Forensic Medical Sciences, Department of Health Sciences, University of Florence, Largo Brambilla 3, 50134 Florence, Italy; martina.focardi@unifi.it (M.F.); beatrice.defraia@unifi.it (B.D.); barbara.gualco@unifi.it (B.G.); costantinoan@aou-careggi.toscana.it (A.C.); rossella.grifoni@unifi.it (R.G.); 2 Laboratory of Personal Identification and Forensic Morphology, Department of Health Sciences, University of Florence, Largo Brambilla 3, 50134 Florence, Italy; vilma.pinchi@unifi.it; 3 General Laboratory, Azienda Ospedaliero-Universitaria Careggi, Largo Brambilla 3, 50134 Florence, Italy; fanellia@aou-careggi.toscana.it (A.F.); biagiolit@aou-careggi.toscana.it (T.B.); costanza.bossi@unifi.it (C.B.); lanzilaol@aou-careggi.toscana.it (L.L.)

**Keywords:** post-mortem interval, vitreous humor (VH), thanatochemistry, forensic pathology, multivariate analysis

## Abstract

**Background/Objectives:** Accurate post-mortem interval (PMI) estimation remains challenging in forensic pathology. Although potassium (K^+^) is the most well-validated single biomarker in vitreous humor (VH), multivariate approaches may enhance precision by capturing the complex cascade of post-mortem biochemical changes. This study aimed to develop and validate a multivariate PMI estimation model incorporating three biochemical markers—potassium, ammonium (NH_4_^+^), and albumin (ALB)—in vitreous humor using automated clinical chemistry platforms for practical forensic application. **Methods:** Vitreous humor samples from 38 autopsy cases with documented PMIs (39.5–285 h; mean, 105.5 h) were analyzed for K^+^ (Cobas C8000), NH_4_^+^ (Cobas C8000), and ALB (Immage 800 nephelometry). Univariate and multivariate regression analyses were performed, with the residual standard error (RSE) as the primary measure of accuracy. Model validation was conducted by back-calculating PMI in four samples completely distinct from the training cohort. **Results:** All three analytes demonstrated strong individual correlations with PMI (R^2^: K^+^ = 0.88, ALB = 0.78, NH_4_^+^ = 0.69; all *p* < 0.001). The multivariate regression model [PMI = 40.25[Alb] + 0.01573[NH_4_^+^] + 5.339[K^+^] − 53.032] yielded an RMSE of ±15.5 h (MSE = 240.25 h^2^), outperforming potassium-only models (RMSE = ±22.6 h). Although NH_4_^+^ showed limited statistical significance in the multivariate model (*p* = 0.128), its inclusion improved overall predictive accuracy. External validation in an independent cohort of four subjects (distinct from the 38 subjects in the training set) demonstrated a mean absolute error (MAE) of 20.4 h. **Conclusions:** The multivariate approach combining K^+^, NH_4_^+^, and ALB in VH improves PMI estimation accuracy compared with single-marker methods. The use of automated clinical chemistry platforms enhances reproducibility and facilitates practical implementation in forensic laboratories.

## 1. Introduction

Estimation of the post-mortem interval (PMI) constitutes one of the most challenging yet crucial tasks in forensic pathology and medicolegal death investigation [1,2]. Accurate PMI determination provides essential temporal information for reconstructing events surrounding death, establishing timelines in criminal investigations, and corroborating or refuting witness statements and alibis [3]. Despite decades of research, PMI estimation remains imprecise, particularly when traditional physical signs become unreliable [4,5]. Vitreous humor (VH) has emerged as the biological fluid of choice for biochemical analysis of the cause of death [6] and for PMI estimation due to its numerous advantageous properties [7,8]. Its anatomical isolation within the globe protects it from environmental contamination and microbial invasion for longer than other body fluids [9]. Its relatively stable composition and minimal cellular content reduce enzymatic degradation, while the ease of collection through simple aspiration makes VH sampling practical in routine autopsy practice [10,11]. Furthermore, VH undergoes predictable post-mortem biochemical changes that can be quantified and correlated with the time since death [12,13].

Among biochemical markers in vitreous humor, potassium (K^+^) has been the most extensively studied and validated for PMI estimation [14,15,16]. The post-mortem rise in vitreous K^+^ results from cellular membrane breakdown and passive diffusion from surrounding ocular tissues, producing a relatively linear increase over time [17,18]. Numerous studies have established regression equations relating vitreous K^+^ concentration to PMI, with reported accuracies typically ranging from ±20 to ±30 h [19,20,21]. Recent meta-analytic evidence confirms K^+^ as a reliable PMI biomarker, with a strong correlation (ρ ≈ 0.69) across diverse study populations [22].

However, reliance on a single biomarker has inherent limitations. Biological variability, analytical uncertainty, and confounding factors such as ambient temperature, cause of death, and antemortem pathophysiology can substantially affect potassium kinetics [23,24,25]. Cold storage prior to autopsy alters regression estimates, with K^+^ tending to overestimate PMI under refrigeration conditions [26]. The manner of death—particularly cardiovascular events, sepsis, and metabolic disorders—influences vitreous biochemistry and may introduce systematic bias [27,28].

Multivariate approaches incorporating multiple biochemical markers may address these limitations by capturing complementary aspects of post-mortem biochemical cascades [29,30]. Ammonium (NH_4_^+^) accumulates in VH through protein deamination and bacterial metabolism, following a temporal pattern distinct from that of potassium [31,32]. The post-mortem rise in NH_4_^+^ is attributed to multiple molecular mechanisms, including proteolysis and amino acid deamination, enzymatic breakdown of nitrogenous tissue components (particularly glutamine and glutamate), and, to a lesser extent, microbial metabolism in cases with prolonged PMI or compromised tissue preservation.

Albumin (ALB), the most abundant plasma protein, increases in vitreous humor post-mortem due to blood–ocular barrier breakdown and passive diffusion from the choroidal circulation [33,34]. Previous work from our group demonstrated that combining ALB with K^+^ improved PMI estimation accuracy compared with potassium alone [9].

Recent advances in analytical chemistry have enhanced the feasibility of multimarker approaches. Automated clinical chemistry platforms offer high-throughput, standardized analysis with excellent precision and minimal sample requirements [22,35]. Capillary electrophoresis enables the simultaneous measurement of multiple ionic species in small vitreous volumes [36]. Nuclear magnetic resonance (NMR) metabolomics and mass spectrometry-based approaches have revealed complex metabolic fingerprints that change systematically with PMI [37,38]. Machine learning algorithms applied to untargeted metabolomic data show promise for improving PMI prediction, particularly during the early post-mortem period [39,40].

Despite these technological advances, practical implementation in routine forensic casework requires validated, reproducible methods using accessible instrumentation [41]. Most forensic laboratories have access to automated clinical chemistry analyzers but lack specialized metabolomics platforms [42]. Therefore, targeted multimarker panels measured on standard clinical instruments represent an attractive compromise between analytical sophistication and practical utility in routine casework [43,44].

The present study aimed to develop and validate a multivariate PMI estimation model combining three biochemical markers—K^+^, NH_4_^+^, and ALB—in VH. We hypothesized that this multimarker approach would improve PMI estimation accuracy compared with single-marker models. All analyses were performed using automated clinical chemistry platforms routinely available in hospital laboratories, facilitating potential translation into forensic practice. We also sought to characterize the individual contribution of each marker and assess the model’s performance across different PMI ranges.

## 2. Materials and Methods

### 2.1. Case Selection and Sample Collection

This study was conducted at the Forensic Science Section, University of Florence, Italy, following approval by the institutional ethics committee (Register Number 12319_bio). Vitreous humor samples were collected from 38 consecutive autopsy cases during the 2023–2024 study period. The cohort was derived from 300 screened autopsies. Of these, 58 cases met the preliminary eligibility criteria, whereas 20 were excluded because of inadequate vitreous humor volume, ocular trauma, advanced putrefaction, or unavailable/unreliable information regarding the time of death.

Cases were selected based on the following inclusion criteria: (1) documented PMI; (2) intact globes without evidence of trauma or decomposition; (3) sufficient VH volume (≥1.5 mL per eye) for complete biochemical analysis; and (4) autopsy performed within 12 days of death.

The exclusion criteria were as follows: (1) signs of decomposition beyond greenish putrefactive staining; (2) ocular trauma or pathology affecting vitreous composition; (3) prolonged cardiopulmonary resuscitation (>30 min); (4) uncertain PMI; and (5) bodies stored at temperatures above 10 °C prior to autopsy.

PMI was calculated as the interval between the documented time of death and the time of vitreous humor collection during autopsy. PMIs ranged from 39.5 to 285 h (mean, 105.5 ± 58.3 h; median, 95.0 h).

VH was collected by the same examiner through direct aspiration from both eyes using sterile 5 mL syringes fitted with 21-gauge needles, according to a previously described method [9]. Samples from both eyes were pooled to minimize intra-individual variability [45]. The VH was immediately transferred to sterile polypropylene tubes and centrifuged at 3000 rpm for 10 min at 4 °C to remove cellular debris and particulate matter. The supernatant was divided into aliquots and stored at −80 °C until analysis.

### 2.2. Biochemical Analysis

All biochemical analyses were performed at the General Laboratory, Azienda Ospedaliero-Universitaria Careggi, Florence, using automated clinical chemistry platforms routinely employed for diagnostic testing.

K^+^ measurement: Vitreous K^+^ concentration was determined by indirect potentiometry using the Cobas C8000 modular analyzer (Roche Diagnostics, Basel, Switzerland). This ion-selective electrode (ISE) method measures K^+^ activity in diluted samples. The analytical measuring range was 2.0–20.0 mmol/L, with an intra-assay coefficient of variation (CV) < 1.5% and an inter-assay CV < 2.0%. Samples with K^+^ concentrations exceeding 20.0 mmol/L were diluted 1:2 with isotonic saline and re-analyzed, with results corrected for dilution.

NH_4_^+^ measurement: NH_4_^+^ concentration was measured enzymatically on the Cobas C8000 analyzer using the glutamate dehydrogenase (GLDH) method. In this assay, GLDH catalyzes the reductive amination of 2-oxoglutarate with NH_4_^+^ and NADPH to form glutamate and NADP+. The concentration of NADP+ formed is directly proportional to the ammonia concentration and is determined by measuring the decrease in absorbance.

ALB measurement: ALB was quantified by immunonephelometry using the Immage 800 analyzer (Beckman Coulter, Brea, CA, USA). This method measures light scattering resulting from antigen–antibody complex formation. Polyclonal anti-human albumin antibodies were used, with turbidimetric detection at 340 nm. The measuring range was 0.05–6.0 g/L, with an intra-assay CV < 2.5% and an inter-assay CV < 3.5%.

All analyses were performed in duplicate, and mean values were used for statistical analysis. Quality control was ensured through daily calibration and analysis of commercial quality control materials at two concentration levels. Samples were thawed only once, immediately before analysis, to avoid freeze–thaw artifacts.

### 2.3. Statistical Analysis

Statistical analyses were performed using SPSS version 27.0 (IBM Corporation, Armonk, NY, USA) and R version 4.2.1 (R Foundation for Statistical Computing, Vienna, Austria). Descriptive statistics were calculated for all variables, including the mean, standard deviation, median, and range. The Shapiro–Wilk test was used to assess the normality of the distributions.

Univariate analysis: Linear regression analysis was performed for each biochemical marker individually against PMI. Pearson correlation coefficients (r) and coefficients of determination (R^2^) were calculated to assess the strength of the associations. The root mean squared error (RMSE) was used as the primary measure of predictive accuracy, alongside the mean squared error (MSE) and mean absolute error (MAE), which represent the average discrepancies between observed and predicted PMI values.

Multivariate analysis: Multiple linear regression was performed with PMI as the dependent variable and potassium, ammonium, and albumin as independent variables. The multivariate model was constructed using the enter method, with all three markers included simultaneously. Model assumptions were verified through examination of residual plots, variance inflation factors (VIFs) to assess multicollinearity, and the Durbin–Watson statistic to assess residual independence.

Regression coefficients, standard errors, t-statistics, and *p*-values were calculated for each predictor. Overall model fit was assessed using R^2^, adjusted R^2^, and RSE. The F-statistic was used to test overall model significance. To evaluate the practical applicability of the regression equations, 95% prediction intervals were computed to quantify the uncertainty associated with individual PMI estimates.

Model validation: Model performance was validated using a leave-one-out cross-validation approach for the main dataset. External validation was conducted using a fully independent validation set consisting of 4 cases that were distinct from the original training cohort of 38 individuals. For these validation cases, PMI was back-calculated using the derived multivariate equation, and absolute errors were computed as the differences between the calculated and actual PMI values.

Statistical significance was set at α = 0.05 for all tests. Confidence intervals (95% CI) were calculated for regression coefficients and predicted values.

## 3. Results

### 3.1. Study Population and Sample Characteristics

The study included 38 cases (26 males and 12 females) with a mean age of 62.4 ± 16.8 years (range, 28–89 years). Regarding causes of death, cardiovascular disease was the primary etiology, accounting for 36.8% (*n* = 14) of all cases, with a predominant distribution in males (*n* = 10; 71.4%) compared with females (*n* = 4, 28.6%). Traumatic injuries constituted the second-most frequent cause of death (*n* = 8; 21.1%), with 75.0% of cases occurring in males (*n* = 6) and 25.0% in females (*n* = 2). Intoxications were identified in six cases (15.8%), comprising four males (66.7%) and two females (33.3%). Asphyxia was observed in four cases (10.5%), with equal distribution between the sexes (*n* = 2 each, 50.0%). Finally, the remaining causes of death, grouped under the category ‘other causes’, comprised six cases (15.8%): four males (66.7%) and two females (33.3%).

The post-mortem interval ranged from 39.5 to 285 h (mean, 105.5 ± 65.6 h; median 93.0 h), with most cases clustered between 48 and 168 h (2–7 days). The Shapiro–Wilk test indicated a distribution with slight positive skewness (W = 0.852, *p* < 0.001). All vitreous samples were clear, with no evidence of significant contamination or hemorrhage.

### 3.2. Biochemical Marker Concentrations

Descriptive statistics for all three biochemical markers and PMI are presented in Table 1. Potassium concentrations ranged from 9.9 to 39.5 mmol/L (mean, 20.5 ± 7.3 mmol/L), exhibiting the expected progressive increase with PMI. Ammonium concentrations ranged from 510 to 2568 μmol/L (mean, 1376.7 ± 491.2 μmol/L), displaying considerable inter-individual variability. Albumin concentrations ranged from 0.18 to 1.98 g/L (mean, 0.68 ± 0.53 g/L), with a progressive increase correlated with PMI.

The Shapiro–Wilk test indicated significant departures from normality for albumin and potassium (W = 0.834, *p* < 0.001; W = 0.935, *p* = 0.028, respectively), with both variables exhibiting slight positive skewness. Conversely, ammonium concentrations exhibited a borderline normal distribution (W = 0.946, *p* = 0.064). Despite its statistical behavior in the model, ammonium remained strongly affected by the overall skewness of the dataset; therefore, Spearman’s rank correlation was preferred (Table 2).

### 3.3. Univariate Regression Analysis

Individual linear regression models were developed for each biochemical marker (Table 3).

Potassium showed the strongest relationship with PMI (R^2^ = 0.88, RSE = ±22.6 h), consistent with its established role as the gold-standard single marker. The regression equation was

**PMI (h) = 8.44 × [K^+^] − 67.6**

Albumin demonstrated a robust correlation with PMI (R^2^ = 0.78, RSE = ±30.2 h), with the following regression equation:

**PMI (h) = 108.63 × [Albumin] + 31.36**

Ammonium showed the weakest individual correlation (R^2^ = 0.69, RSE = ±36.0 h), with the following regression equation:

**PMI (h) = 0.1108 × [NH_4_^+^] − 47.099**

All three models showed statistically significant relationships with PMI (*p* < 0.001), although their predictive accuracies varied, as reflected by their RSE values.

### 3.4. Chronological Stratification and Model Comparison

To evaluate the stability of the predictive framework across different chronological stages, the cohort was divided into three post-mortem intervals: <72 h (*N* = 12), 72–144 h (*N* = 20), and >144 h (*N* = 6). Separate multivariate ordinary least squares (OLS) regressions were generated for each interval.

Formal comparison via ANCOVA demonstrated that the slopes of the derived sub-equations were not statistically equivalent (*p* < 0.01 for all interaction terms). In the early interval (<72 h), the regression fit was weak (R^2^ = 0.254), reflecting a physiological lag phase in autolytic leakage. Conversely, robust linear trends emerged in the intermediate (R^2^ = 0.827) and late intervals (R^2^ = 0.985), driven by extensive post-mortem cell lysis. These significant structural differences between the time-restricted models confirm the non-linear, multi-phasic nature of vitreous humor degradation over extended post-mortem periods and justify the implementation of our tempered macro-regional model rather than fragmented sub-interval equations.

### 3.5. Multivariate Regression Analysis

Multiple linear regression incorporating all three markers yielded a significantly improved model compared with univariate approaches (Table 4). The multivariate regression equation was

**PMI (h) = 40.25 × [Albumin] + 0.01573 × [NH_4_^+^] + 5.339 × [K^+^] − 53.032**

This model achieved R^2^ = 0.91 and RMSE = ±15.5 h (MSE = 240.25 h^2^), representing a 31% reduction in prediction error compared with the potassium-only model. The model also demonstrated a mean absolute error (MAE) of 16.8 h. The overall model was highly significant (R^2^ = 0.91; adjusted R^2^ = 0.90; RMSE = 15.5 h; MSE = 240.25 h^2^; MAE = 16.8 h; F = 118.3; *p* < 0.001).

Examination of variance inflation factors (VIFs) revealed minimal multicollinearity between predictors (all VIFs < 2.0), indicating that the markers provided complementary information. Residual analysis showed an approximately normal distribution with no systematic patterns, confirming that model assumptions were met.

**Figure 1:**
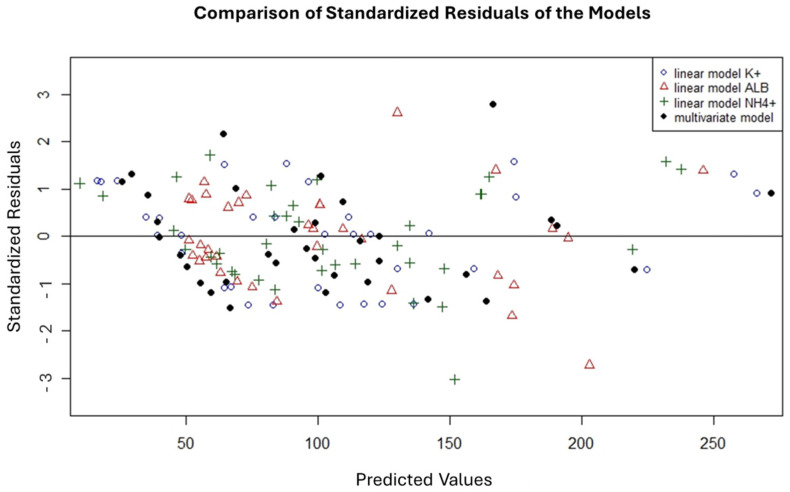
Comparison of standardized residuals of the multivariate regression model.

Notably, although ammonium showed marginal statistical significance in the multivariate model (*p* = 0.128), its inclusion improved overall model fit and reduced the RMSE, as evidenced by the narrower distribution of standardized residuals compared with univariate models (Figure 1). This suggests that ammonium contributes useful information despite not reaching conventional significance thresholds, possibly due to its distinct temporal kinetics, which capture separate phases of post-mortem change.

### 3.6. Model Validation

Leave-one-out cross-validation of the multivariate model yielded a cross-validated R^2^ of 0.91 and a mean absolute error of 16.8 h, demonstrating robust internal validity with minimal overfitting.

External validation was performed on an independent set of four cases (V1–V4) that were entirely external to the original development cohort of 38 individuals (Table 5). These validation cases spanned PMIs from 48 to 192 h. The multivariate model predicted PMI with a mean absolute error (MAE) of 20.4 h (range, 7.98–32.9 h), consistent with the RMSE derived from the training dataset.

Comparison of predicted versus actual PMI values showed good agreement across the PMI range, with slightly larger absolute errors at longer PMIs, although relative errors remained consistent.

## 4. Discussion

This study demonstrates that a multivariate approach combining K^+^, ALB, and NH_4_^+^ in VH significantly improves PMI estimation accuracy compared with conventional single-marker methods. The developed model achieved a residual standard error of ±15.5 h, representing a 31% improvement over potassium-only models. Importantly, all analyses were performed using automated clinical chemistry platforms routinely available in hospital laboratories, facilitating practical implementation in forensic settings and enabling a rapid response to investigators. These methods can be further supplemented and integrated with more advanced techniques that are not available in all laboratories and are difficult to apply in routine practice [39,46]. The superiority of multivariate over univariate models aligns with emerging evidence that multiple biochemical markers capture complementary aspects of post-mortem change [37,38]. The integration of 95% prediction intervals provides a robust safety margin for investigators, shifting interpretation from a single point estimate of the time since death to a scientifically grounded temporal window. Recent metabolomic studies using NMR spectroscopy have demonstrated that multivariate models incorporating metabolic profiles predict PMI more accurately than K^+^ alone, particularly in the early to intermediate post-mortem periods [46]. Locci et al. reported that a combined NMR metabolomics and K^+^ model achieved prediction errors of 6.9 h for PMI < 24 h and 7.4 h for PMI 24–48 h in an animal model, substantially outperforming single-marker approaches [47]. Similarly, Løber et al. applied machine learning to untargeted metabolomic data and achieved high cross-validated accuracy using a small panel of approximately 15 metabolites selected through feature-selection algorithms [39]. These sophisticated approaches demonstrate the potential of multivariate analysis but require specialized instrumentation and expertise not readily available in most forensic laboratories. Our targeted three-marker panel represents a practical middle ground, capturing multivariate information while using accessible analytical platforms. The use of automated clinical chemistry analyzers offers several advantages, including standardized methodology, high throughput, excellent precision, minimal sample requirements, and widespread availability [48]. These characteristics facilitate quality assurance, inter-laboratory comparability, and potential implementation in routine forensic practice [49].

K^+^ demonstrated the strongest individual correlation with PMI (R^2^ = 0.88), confirming its status as the most well-validated single biomarker in UV.

However, K^+^ kinetics are influenced by multiple confounding factors. Ambient temperature significantly affects the rate of potassium increase, with cold storage prior to autopsy tending to overestimate PMI [26,50]. The cause of death also influences vitreous K^+^, particularly in cases involving cardiovascular events, electrolyte disturbances, or renal dysfunction [27,28]. Analytical methodology introduces additional variability, with ion-selective electrode methods showing higher measurement uncertainty than microwave-induced plasma optical emission spectrometry (MIP-OES) [51].

ALB showed a robust correlation with PMI (R^2^ = 0.78) and contributed significantly to the multivariate model. Our previous work demonstrated that combining ALB with K^+^ Improved PMI estimation compared with K^+^ alone [9], a finding corroborated by the present study. ALB may capture different aspects of post-mortem change than K^+^, including vascular permeability alterations and tissue autolysis [52]. In contrast to our previous study [9], which indicated that ALB exhibited an apparently exponential pattern, the findings of the present study suggest a linear trajectory, likely attributable to the higher frequency of sampling at advanced post-mortem intervals. ALB measurement by immunonephelometry is highly precise and widely available in clinical laboratories. However, ALB concentrations may be influenced by antemortem hypoalbuminemia, ocular inflammation, and vitreous hemorrhage [53]. Despite these potential confounders, albumin’s complementary temporal pattern enhances multivariate model performance.

NH_4_^+^ exhibited the weakest individual correlation (R^2^ = 0.69) but contributed to improved multivariate model fit. Musile et al. developed a microfluidic paper-based device for rapid NH_4_^+^ analysis in VH, demonstrating its potential as a field-deployable PMI marker [54].

In our multivariate model, NH_4_^+^ showed marginal statistical significance (*p* = 0.128) but contributed to a reduced RSE. This suggests that NH_4_^+^ provides useful information despite not reaching conventional significance thresholds, possibly due to its distinct temporal kinetics or sensitivity to factors not captured by potassium and albumin. The relatively high inter-individual variability in NH_4_^+^ concentrations may reflect differences in protein content, bacterial activity, or metabolic state at death [55].

Analytical uncertainty remains relevant. In our study, we used an ion-selective electrode methodology on the Cobas C8000 platform, representing the standard approach in clinical laboratories but introducing greater uncertainty than spectrometric methods.

Measurement uncertainty propagates through regression equations to produce PMI prediction intervals [56]. The residual standard error of ±15.5 h in our multivariate model represents the combined effect of biological variability, analytical imprecision, and model limitations.

Quality assurance is essential for reliable results. We employed daily calibration, duplicate analyses, and quality control materials at multiple concentration levels. However, VH lacks certified reference materials, and matrix effects may differ from aqueous calibrators [57]. The development of matrix-matched quality control materials and proficiency testing programs would enhance standardization across forensic laboratories [58].

Multiple biological and environmental factors influence VH biochemistry and must be considered in PMI interpretation [23,24,25]. Temperature-adjusted models or separate equations for different storage conditions may be necessary [59]. In our study, we did not consider environmental variables, as the bodies were exposed to uncontrolled ambient temperatures for less than six hours after death. Although this is a short period, it nonetheless represents a limitation that may affect the results.

The cause of death influences vitreous biochemistry through multiple mechanisms [27,28]. Our study population included diverse causes of death, potentially increasing model robustness across different scenarios, although subgroup analyses were limited by sample size.

Intra-individual variability has been documented in recent studies [45]. We pooled vitreous fluid from both eyes to minimize this variability, consistent with common forensic practice. Moreover, VH was collected by the same examiner to reduce inter-examiner variability.

Antemortem pathophysiology may affect baseline concentrations of biochemical markers. Chronic kidney disease, liver cirrhosis, and electrolyte disturbances alter potassium homeostasis [60]. Ideally, PMI models should account for antemortem medical history, although this information is often unavailable in forensic contexts.

While our targeted three-marker panel improves upon single-marker methods, emerging technologies offer even greater potential. NMR metabolomics provides comprehensive metabolic fingerprints that change systematically with PMI [47]. However, NMR requires specialized instrumentation, expertise, and data analysis capabilities that are not available in most forensic laboratories [37].

Mass spectrometry-based metabolomics offers high sensitivity and specificity for metabolite identification and quantification. Untargeted approaches identify hundreds of metabolites, enabling the discovery of novel PMI markers [61]. However, metabolomics workflows are time-consuming, expensive, and require sophisticated bioinformatics [62].

Chemometric approaches applied to alternative analytical techniques show promise. Risoluti et al. used thermogravimetric analysis combined with chemometric classification to predict PMI in contaminated vitreous specimens, achieving ≥70% accuracy [63]. Such approaches may complement biochemical analysis when sample quality is compromised.

Artificial intelligence and machine learning applied to multivariate biochemical data may further improve PMI prediction. Neural networks and ensemble methods can model complex non-linear relationships and interactions between markers [64]. However, these approaches require large training datasets and careful validation to avoid overfitting [65].

Our targeted multimarker approach using clinical chemistry platforms represents a pragmatic compromise, capturing multivariate information with accessible technology. As metabolomics and machine learning mature, the integration of discovery-based approaches with targeted validation may yield optimized marker panels for routine forensic use [66].

Several factors facilitate the practical implementation of our multivariate model, for which analytical accessibility is essential. The above-mentioned platforms are widely available in hospital laboratories, and many forensic institutes have access to these or similar instruments [48]. Sample requirements are modest—approximately 0.5 mL of VH suffices for all three analyses. Turnaround time is rapid: results are typically available within 2–3 h, compatible with autopsy workflows. Quality assurance is facilitated by standardized methods, automated calibration, and commercial quality control materials, the costs of which are reasonable for daily forensic work. Therefore, this method appears to be readily implementable across forensic medicine institutes, providing rapid results and enabling the identification and selection of specific cases for subsequent, more in-depth analyses (e.g., metabolomics and proteomics).

An implementation workflow would involve the following: (1) standardized vitreous collection during autopsy; (2) centrifugation and aliquoting; (3) analysis on clinical chemistry platforms; (4) calculation of PMI using the regression equation; and (5) interpretation considering the case context and potential confounders. Integration of the equation into laboratory information systems could automate calculation and reporting.

Limitations for implementation include the need for validation in individual laboratories, the development of quality control procedures specific to VH, and the training of personnel. Inter-laboratory validation studies would establish the generalizability of the model across different populations, analytical platforms, and environmental conditions [58].

Several limitations warrant acknowledgment. The sample size (*n* = 38) is modest, although comparable to that of many published PMI studies. Larger cohorts would enable more robust validation, subgroup analyses by cause of death or storage conditions, and investigation of non-linear relationships. The PMI range was limited to 39.5–285 h, with most cases in the 48–168 h range. Validation in the very early (<24 h) and very late (>2 weeks) PMI ranges is needed.

All cases were from a single Italian institution, potentially limiting generalizability to other geographic regions, climates, or populations. Multicenter validation studies are essential. Confounding factors were not systematically controlled. Although we documented storage temperature and cause of death, we did not adjust the models for these variables due to the limited sample size. Future studies should develop stratified or adjusted models.

External validation was limited to four cases. Larger independent validation cohorts are needed to confirm model performance and establish prediction intervals. Mechanistic understanding of the interrelationships between markers and their responses to confounding factors remains incomplete. Controlled studies investigating temperature, cause of death, and antemortem pathophysiology would enhance interpretability.

Several research directions would advance multivariate PMI estimation. Expanded marker panels incorporating additional biochemical analytes (e.g., hypoxanthine and creatinine) or metabolomic profiles may further improve accuracy [66]. Machine learning approaches applied to multivariate biochemical data could model complex non-linear relationships and interactions [64,65].

Integration with physical methods in Bayesian frameworks could provide probabilistic PMI estimates with quantified uncertainty [67].

Standardization initiatives, including inter-laboratory validation, proficiency testing, and the development of reference materials, would facilitate widespread implementation [58]. Point-of-care devices for rapid field analysis could enable PMI estimation at death scenes, informing investigative priorities [54]. Longitudinal studies with serial sampling would elucidate temporal kinetics and inter-individual variability [45].

Combining targeted biochemical markers with untargeted metabolomics, proteomics, or transcriptomics may reveal novel markers and mechanistic insights [37,38]. Artificial intelligence applied to multimodal data (biochemical, imaging, and environmental) could provide comprehensive PMI estimation systems [68].

## 5. Conclusions

This study demonstrates that multivariate analysis of K^+^, NH_4_^+^, and ALB in VH improves post-mortem interval estimation accuracy compared with conventional single-marker approaches. The developed model achieved a residual standard error of ±15.5 h, representing a 31% improvement over K^+^-only methods. All analyses were performed using automated clinical chemistry platforms routinely available in hospital laboratories, facilitating practical implementation in forensic settings.

The complementary temporal kinetics of the three markers captured different aspects of post-mortem biochemical cascades, enhancing predictive accuracy across the PMI range studied (39.5–285 h). Although K^+^ remained the strongest individual predictor, the addition of ALB and NH_4_^+^ provided incremental information that reduced prediction error.

The practical advantages of the multivariate approach include (1) the use of accessible analytical platforms, (2) modest sample requirements, (3) rapid turnaround time, (4) standardized methodology supporting quality assurance, and (5) reasonable cost. These characteristics support potential translation into routine forensic practice.

However, several considerations are essential for appropriate application. Confounding factors, including storage temperature, cause of death, and antemortem pathophysiology, may influence marker concentrations and should be considered in PMI interpretation. Validation in larger, multicenter cohorts is needed to establish generalizability across diverse populations and environmental conditions. Integration with other PMI estimation methods within comprehensive frameworks may provide optimal accuracy.

Moreover, the model is not validated for very early PMI estimation (<24 h), advanced decomposition beyond the studied range, or cases involving major environmental or biological conditions not represented in the cohort.

Future research should focus on (1) expanded validation in larger cohorts spanning wider PMI ranges; (2) investigation of confounding factors and development of adjusted models; (3) exploration of additional markers and advanced analytical approaches; (4) standardization initiatives to facilitate inter-laboratory comparability; and (5) integration of biochemical methods with physical and environmental indicators within probabilistic frameworks.

The multivariate approach combining K^+^, NH_4_^+^, and ALB in VH represents a promising model for post-mortem interval estimation, offering improved accuracy through an accessible methodology, but it requires prospective multicenter validation. With appropriate validation and consideration of confounding factors, this approach has the potential to enhance the precision and reliability of PMI determination in forensic practice as an adjunct tool rather than as a standalone determinant.

## Figures and Tables

**Table 1:** Descriptive statistics of PMI and biochemical markers in vitreous humor (*n* = 38).

Parameter	Mean ± Sd	Median	Range	95% CI
**PMI (h)**	105.5 ± 65.6	93	39.5–285.0	83.6–127.4
**Potassium (mmol/L)**	20.5 ± 7.3	19.8	9.9–39.5	18.1–22.9
**Ammonium (μmol/L)**	1376.7 ± 491.2	1258.9	510.0–2568.0	1212.7–1540.6
**Albumin (g/L)**	0.68 ± 0.53	0.5	0.18–1.98	0.50–0.86

**Table 2:** Spearman’s rank correlation matrix (rho) between actual PMI and vitreous biomarkers (all *p*-values < 0.001).

Parameter	PMI (TSD H)	Potassium (K^+^)	Ammonium (NH_4_^+^)	Albumin
PMI (TSD H)	1	0.86	0.694	0.839
Potassium	0.86	1	0.617	0.663
Ammonium	0.694	0.617	1	0.704
Albumin	0.839	0.663	0.704	1

**Table 3:** Univariate linear regression models for PMI estimation.

Marker	Regression Equation	R^2^	RMSE (h)	MSE (h^2^)	MAE (h)	*p*-Value
Potassium	PMI = 8.44[K^+^] − 67.6	0.88	±22.6	510.76	18.1	<0.001
Albumin	PMI = 108.63[Alb] + 31.36	0.78	±30.2	912.04	24.2	<0.001
Ammonium	PMI (h) = 0.1108[NH_4_^+^] − 47.099	0.69	±36.0	1296.00	28.8	<0.001

**Table 4:** Multivariate linear regression model for PMI estimation.

Coefficient	Estimate	STD. Error	T-Value	*p*-Value	95% CI Lower	95% CI Upper	VIF
**Intercept**	−53.032	10.806	−4.908	<0.001	−75.017	−31.046	—
**Albumin**	40.250	9.661	4.166	<0.001	20.595	59.906	**1.70**
**Ammonium (NH_4_^+^)**	0.01573	0.01006	1.563	0.128	−0.00476	0.03622	**1.56**
**Potassium (K^+^)**	**5.339**	**0.641**	**8.330**	**<0.001**	**4.035**	**6.643**	**1.76**

**Table 5:** External validation results.

Sample	Potassium (K^+^) (mmol/L)	Albumin (g/L)	Ammonium (NH_4_^+^) (µmol/L)	Actual PMI (h)	Predicted PMI (h)	Prediction Error (h)	Relative Error (%)
V1	20.2	0.623	1820	98	108.6	10.6	11%
V2	24	0.8264	1430.8	98	130.9	32.9	34%
V3	17.8	0.8554	1828	74.92	105.2	30.28	40%
V4	15.6	0.8255	1231.2	74.92	82.9	7.98	11%
Mean	—	—	—	—	—	+20.4	24%

## Data Availability

The original contributions presented in this study are included in the article. Further inquiries can be directed to the corresponding author.
